# Equity and inclusivity in research: co-creation of a digital platform with representatives of marginalized populations to enhance the involvement in research of people with limited literacy skills

**DOI:** 10.1186/s40900-021-00313-x

**Published:** 2021-10-05

**Authors:** Christine Loignon, Sophie Dupéré, Caroline Leblanc, Karoline Truchon, Amélie Bouchard, Johanne Arsenault, Julia Pinheiro Carvalho, Alexandrine Boudreault-Fournier, Sylvain Aimé Marcotte

**Affiliations:** 1 Department of Family and Emergency Medicine, Faculty of Medicine and Health Sciences, Université de Sherbrooke, Longueuil, QC Canada; 2 Faculty of Nursing, Université Laval, Quebec, QC Canada; 3 Department of Social Sciences, Université du Québec en Outaouais, Gatineau, QC Canada; 4 Villeray Literacy Center – La Jarnigoine, Montreal, QC Canada; 5 Atout-Lire Literacy Center, Quebec, QC Canada; 6 Le Tour de Lire Literacy Center, Montreal, QC Canada; 7 Department of Anthropology, University of Victoria, Victoria, BC Canada

**Keywords:** Patient and public involvement, Literacy, Co-creation, Equity in research, Visual methods, Popular education, Participatory research, Inclusivity

## Abstract

To improve health equity, as well as equity in research, community-engaged research and participatory research needs to be inclusive. Equity in health research refers to the principle that anyone affected by research or who can benefit from its outcomes should have equal opportunities to contribute to it. Many researchers advocate the importance of promoting equity in research and engage in processes that foster the research involvement of lay persons, patients, and community members who are otherwise “absent” or “silent”. Still, people with limited literacy skills who experience unwarranted structural barriers to healthcare access have little involvement in research. Low literacy is a major barrier to equity in health research. Yet there exist approaches and methods that promote the engagement in research of people with literacy challenges. Building on our previous research projects conducted with community members using participatory visual and sound methods (participatory mapping, photovoice, digital storytelling, etc.), we embarked on the co-creation of a digital platform in 2017. Our aim in this commentary is to report on this co-creation experience that was based on a social justice-oriented partnership. The development of the online platform was overseen by a steering committee made up of workers from community organizations involved with people with limited literacy skills, students, and researchers. In the development process, the co-creation steps included a literature review, informal interviews with key informants, and discussion and writing sessions about format and content. After numerous challenges raised and addressed during co-creation, the Engage digital platform for engagement in research went live in the winter of 2020. This platform presents, on an equal footing, approaches and methods from academic research as well as from the literacy education community engaged with people with limited literacy skills.

## Plain English Summary

People with limited literacy skills are often excluded from health research. Engaging patients and community members with limited literacy in research requires tailored approaches and methods that have been tried and tested with them. In 2017, building on an existing partnership between researchers well-versed in using participatory visual and sound methods and community partners, we undertook the co-creation of a digital platform. Our aim was to empower both academic researchers and community researchers and partners (lay persons, clinicians, stakeholders, community organizations) to engage in research projects with people with limited literacy skills. The result was a digital platform (http://www.engageplus.com) comprising several modules and resources available in French and English and accessible on the Web. In this commentary, we share our experience in co-creating this digital platform and discuss the facilitators and challenges encountered.

## Background

The benefits of the science of engagement are increasingly recognized [1, 2]. Involving people in research who are in socially vulnerable situations is a proven avenue for developing relevant knowledge to better adapt health interventions and policies aimed at reducing social inequalities in health [3, 4]. Participatory research conducted with disadvantaged people has documented positive outcomes for these populations (better health results, increased health literacy, improved access to health services) [5] as well as more broadly for the community and the intervention [6–8]. However, marginalized people remain little involved in research and are too often confined to a consultative role rather than full and active participation [9, 10]. Some disadvantaged groups are relegated to the status of a "weak public" and thus to having little presence in research and little influence with health care researchers or decision-makers [11].

Engaged research enables the active, equitable, and real participation of marginalized persons, with a view to avoiding their instrumentalization or a symbolic participation that would primarily serve the objectives of researchers rather than their own. Such engaged research would allow their diverse points of view and experiential knowledge to be expressed and considered [12, 13]. McCoy points out that public participation in research often involves those deemed most competent and disposed to participate because of their education (such as a university degree), financial status (ability to cover expenses prior to reimbursement), and context (e.g. access to transportation) [14]. Because of this, some researchers have raised cautions about the risk of incidental or symbolic (tokenistic) participation in projects involving disadvantaged or marginalized persons [15, 16].

Researchers face many challenges in integrating these populations as active partners or co-researchers. Despite promising initiatives, academic institutions and funding agencies in Canada struggle to ensure a certain representativeness of the Canadian population and promote the inclusion of marginalized people in health research. Several studies have reported difficulties in recruiting these “hard to reach” populations, supporting their participation, gathering their voices, and including them fully in the dissemination of research results [17].

Our work over more than a decade has forged an alliance between two milieus: the academic and the community. This alliance has two broad aims: to overcome prejudices in the health system towards people living in poverty and to empower persons with limited literacy skills through research. As participatory action researchers, through these experiences, we have been called to reflect on our own privileges (as academic researchers and community workers) and their repercussions on our research partnership. Given that our work is conducted in a context of social class differences, we aim for a "de-elitization" of our research process [10]. To achieve this, we use methods tailored to people’s interests, values, and abilities, as well as methods developed by members of oppressed communities to bridge social distance and enable people to reflect on their living and health conditions. These methods, such the merging of knowledge, a method developed by the ATD Fourth World movement [18], and other methods developed in the popular education movement by community workers or educators, must find their place in research. Community-engaged research is conducted in alliance with community members, whose access to decision-makers is limited or non-existent, based on their needs and concerns rather than on those of researchers or clinicians who enjoy privileges and are close to decision-makers.

However, suitable methods are essential. There are several participatory visual and sound methods—such as photovoice, digital storytelling, and walking methods—that are well-suited to people with limited literacy skills, which allow them to express their embodied experience verbally and to share feelings in ways that encourage reflexivity. Participatory visual and sound methods are a promising avenue to better include people in the production and sharing of knowledge [19–27]. Their use stems from a long tradition in social sciences, but they are still rarely employed in health research, despite being very inclusive methods with emancipatory potential. In the following sections, we present our approach to co-creating, with representatives of marginalized populations, the online platform Engage (www.engageplus.org). This platform is designed to promote the active engagement in research of people with limited literacy skills through participatory visual and sound methods and facilitation tools derived from popular education. We describe the co-creation process with partners who were involved as community workers and educators in literacy community organizations and briefly describe the platform. We then discuss some key challenges encountered how these were managed.

## The co-creation process for the Engage digital platform

The Engage project builds upon a research program developed in 2009 with representatives of marginalized populations which engendered several projects using participatory visual and sound methods (photovoice, digital storytelling, etc.). Between 2017 and 2020, three community organizations working in literacy education—Le Tour de Lire, Atout-Lire, and La Jarnigoine—joined forces with our research team to form a research partnership engaged with the community. The goal of this partnership, funded by the Canadian Institutes of Health Research, was to support the research engagement of individuals and communities affected by social inequalities, poverty or precariousness, social exclusion, and low literacy, with a view to better aligning research so as to adapt health and services more closely to their needs and realities. We decided to co-develop an easily accessible virtual portal for all those wishing to use approaches, methods, resources, and tools that encourage active participation in research by people who are not often involved in research, i.e., those with limited literacy skills. More specifically, the Engage website was designed as a portal offering concrete tools to support all people (researchers, students, stakeholders, lay persons, etc.) interested in engaged or participatory research using visual and sound methods, facilitation tools, and engagement methods stemming from the popular education movement (River of Life, problem tree analysis, etc.). It aims to empower members of the public and patients with limited literacy skills as well as health organizations that wish to develop knowledge and skills related to participatory research using visual and sound methods.

The development in co-creation of the Engage digital platform was aimed at supporting the process of engagement in research among people experiencing social exclusion in society. The platform was also aimed at strengthening equity in research and reducing barriers between academic research and the wider community. Presented in the form of a website, it includes information modules on approaches and research methods recognized by both academic researchers and community experts as promoting the participation of people often excluded from more traditional research designs. The modules familiarize community members and researchers with the basics of participatory research and provide tutorials on visual, sound, or experiential methods that can be used to lower the barriers preventing people with limited literacy skills from participating in research.

In co-creating the Engage portal, we completed several steps before going live. First, we formed a steering committee made up of three persons working in community organizations involved with people with limited literacy skills (AB, JA, SAM), two students (JPC, CLe), one in medicine and the other in public health who had a history of social exclusion, and three researchers (SD, CL, KT) with complementary expertise (health promotion, medical sociology, visual anthropology, etc.) and experience in engaged research in the community. This committee met on a regular basis (3x/year between 2017 and 2020) to ensure project follow-up and contribute to the design and drafting of the digital platform sections.

Then, as a foundation for developing a digital platform for conducting research using participatory visual and sound methods, we conducted a narrative literature review on the transformation of health services in partnership with individuals or groups in socially vulnerable situations. The content of the various platform sections was developed based on best practices identified through this literature review. The objective of the literature review was to identify empirical studies conducted in partnership with community members or socially vulnerable individuals in which visual and/or sound methods were used to facilitate the research process. In the literature review process, we: (1) identified the key words with the help of visual/sound methods experts on our team and one outside expert in the science of information; (2) explored multiple databases (Proquest, Medline, CINAHL, ERIC) from 2000 to 2018; (3) selected pertinent articles (46 met our inclusion criteria); and (4) extracted the data into an Excel sheet. Our inclusion criteria were: (1) empirical research using visual and/or sound methods; and (2) engagement of community members living in socially vulnerable situations.

Finally, the third step consisted of five informal interviews with key informants (researchers or experts from community organizations) who had initiated and/or participated in research projects using visual and/or sound methods with marginalized community members. These were conducted in 2018 in French or English by two students and a senior research assistant. Interview guides were developed based on the literature and the expertise of the team. However, those interviews were informal key informant interviews and not semi-structured or structured interviews. The goal was to document the benefits and strengths of these methods of engagement in research.

Table 1 summarizes the benefits of using participatory visual and sound methods, as identified in discussions among our team following the literature review and key informant interviews.

**Table 1:** Advantages of using visual and sound methods to engage patients and community members in research

1	Promotes the involvement of the people concerned even when the concepts are complex
2	Helps to communicate and reflect on sensitive subjects or those for which words are difficult to find
3	Captures aspects of people's everyday lives that would not be as effectively revealed by other research methods that require literacy skills (e.g., questionnaire) or that may be perceived as impersonal (e.g., survey) or intimidating (e.g., interview)
4	Facilitates the re-creation of sensory and emotional events or perceptions
5	Provides an opportunity for people to show how they feel about the experience they are having
6	Empowers the people directly involved to collect the data and report the results
7	Promotes people's reflexivity about their experience, helps them communicate this experience and build their own narrative
8	Allows individuals to develop/reinforce the belief that they are competent to communicate their experience
9	Creates links and helps with dialogue between partners and researchers
10	Leads to a shared understanding between partners and researchers
11	Generates results that partners can relate to
12	Allows partners to take ownership of the results and makes them accountable for the research findings
13	Instead of feeling like objects that are analyzed or from which data are drawn, partners become active co-creators of knowledge
14	Enables partners to advocate positions that can benefit other marginalized people

We then chose a webmaster who would accept to develop the website in a co-creation process. Our partners wanted to be involved in the visual presentation, the platform structure, and the writing of the different modules. CLe was very active as the intermediary between the steering committee and the webmaster, both of whom made clarifications or additions at each stage. CLe reviewed the webmaster’s work as required to incorporate feedback and changes requested by the steering committee. All researchers and community partners, as well as other collaborators external to the steering committee, wrote or were involved in reviewing the content of the modules and tutorials. After numerous challenges raised and addressed during co-creation, the Engage digital platform for engagement in research went live in the winter of 2020. This platform presents, on an equal footing, approaches and methods from academic research (e.g., participatory research and photovoice) and from the literacy education community engaged with people with limited literacy skills. For instance, the REFLECT approach and problem tree analysis are two methods inspired by the awareness-raising approach developed by Paolo Freire and are used in many countries especially in popular education community organizations. Each method is presented as a tutorial that allows users to follow the method step by step and apply it in their research context. The site includes several resources and reference documents to accompany researchers and community partners wishing to use or apply these approaches and methods in their research projects.

## Facilitators and challenges in co-creating a digital engagement platform

Before presenting some of the challenges encountered, we should underscore the key facilitators that supported the co-creation of the platform. The primary facilitator was the fact that our partnership was firmly grounded in strong pre-existing relationships, as we had all been involved together in earlier participatory research projects or research partnership initiatives. The bond of trust had been established among us within the team for several years. Authenticity and respectful dialogue were the hallmarks of our partnership, and so we were one step ahead when we started the steering committee meetings.

The first key challenge concerned the platform’s target audience. We originally wanted this platform to speak directly to people with limited literacy skills so they could become empowered and engaged in research, which was consistent with our community-engaged research approach. According to our team, however, this would have required applying language simplification techniques to all content, integrating an audio player, and creating and adding videos. We discussed extensively this ideal that united us, but eventually agreed that for pragmatic reasons (lack of resources and time) we had to restrict ourselves to taking the first step: offering a digital platform to support engagement that would allow researchers and community members to involve people. The second challenge was the difficulty in avoiding a hierarchy of knowledge, i.e., giving precedence to knowledge from scientific research over knowledge from the field experience of popular education responders. Within the scientific community there is, generally speaking, a lack of recognition or credibility given to the approaches and methods developed by community experts. We discussed these issues at our work meetings, and our community partners, experts in the field of literacy, decided to leap into the breach and take the time to document their practices and write modules and tutorials. They did, however, advise us of their lack of time to do this, given that they were fully devoted to supporting individuals with limited literacy skills within their respective organizations. We agreed to recognize their expertise with a financial reward to facilitate this involvement and that we would be flexible about timelines. On the whole, this has been a positive and mutually rewarding experience for the researchers, students, and community partners.

## Conclusions

Co-creation of the Engage digital platform for engagement in research was intended to fill a gap in practical tools and resources to address the challenges of engaging people with limited literacy skills in research. The platform includes tutorials on participatory visual and sound methods and modules on approaches and facilitation methods that are known to promote the inclusion and diversity of patients’ and community members’ engagement in research. We envision that the platform will continue to be enhanced and that a peer review committee including community members will evaluate proposals from researchers and the public who wish to contribute stories, modules, etc. It would be advisable for the digital platform or a portion of it to be adapted for direct online access by people with limited literacy skills.

## Data Availability

Not applicable.
